# Perceived organizational support and its impact on employee's intention to stay: Dataset from the electronics industry in Vietnam.

**DOI:** 10.1016/j.dib.2024.110428

**Published:** 2024-04-18

**Authors:** Cong Hiep Duong, Yi-Hui Ho

**Affiliations:** a College of Management, Chang Jung Christian University, Tainan, Taiwan; b Faculty of Accounting, Thai Nguyen University of Economics and Business Administration, Thai Nguyen, Vietnam

**Keywords:** Electronics industry, Employee's intention to stay, Job satisfaction, Perceived organizational support, Work-life balance, Vietnam

## Abstract

This dataset investigates the complex interactions between perceived organizational support (POS) and Employee's intention to stay (ITS) in Vietnam's electronics industry, emphasizing the subtleties of job satisfaction (JS) and work-life balance (WLB) against a backdrop of socialist economic principles. The analysis is underpinned by a structured questionnaire distributed among employees across various corporations, including prominent entities like Samsung, Foxconn, and Luxshare, in Vietnam's northern industrial zones. A total of 604 legitimate responses were amassed via a convenience sampling strategy. After meticulous collation and organization, the dataset was subjected to Partial Least Squares Structural Equation Modeling (PLS-SEM) to elucidate the symbiotic relationships among POS, JS, WLB, and ITS. The outcomes obtained from this dataset show the relationship between POS, JS and WLB had a positive and significant impact on ITS. This dataset can offer valuable insights to countries with similar characteristics to Vietnam.

Specifications Table

**Table:** 

Subject	Business, Management and Decision Sciences.
Specific subject area	Human Resource Management, Industrial Electronics, Organizational Behaviour.
Type of data	Table, Figure. Raw.
Data collection	The data collection for this study was facilitated through an online survey deployed via Google Forms, disseminated through emails and social networks between November 2022 and February 2024. The design of the survey incorporated both Convenience and Simple Random Sampling techniques. Respondents completed a detailed questionnaire that included a section of eight demographic-related questions and a larger section of 21 questions that delved into areas such as perceived organizational support (POS), intention to stay (ITS), Job satisfaction (JS), and work-life balance (WLB). The POS construct was measured using an eight-item scale based on the work of Eisenberger, Huntington, Hutchison and Sowa [1]. ITS was evaluated using a five-item scale informed by the research of Bangwal and Tiwari [2], Mrayyan [3], and Nasyira, Othman and Ghazali [4]. The JS construct was assessed with a five-item scale based on Moqbel, Nevo and Kock [5]. Additionally, WLB was appraised using a three-item scale from Haar, Sune, Russo and Ollier-Malaterre [6]. Every construct assessed in the survey utilized a five-point Likert scale for responses. Participants were briefed on the purpose and significance of the study before they could access the survey questions. Eligibility for participation was restricted to individuals currently employed in the electronics industry and at least 18 years old.
Data source location	The research was conducted within the electronics industry located in the northern region Country: Vietnam
Data accessibility	Repository name: Mendeley Data Data identification number: 10.17632/pyjkvgjmfz.2 Direct URL to data: https://data.mendeley.com/datasets/pyjkvgjmfz/2

## 1 Value of the Data

• The dataset provides vital insights into employee's intention to stay in Vietnam's electronics industry.
• A survey examined how perceived organizational support, job satisfaction, and work-life balance influence employee's intention to stay in the electronics industry.
• Policy-makers can use data analysis to improve employee's intention to stay, particularly in the electronics industry.
• The survey design is well-suited for organizational studies across various industries
• This data is precious for researchers looking to explore employee behavior. The dataset's results are particularly vital for comparative studies across different countries or industries.

## 2 Background

The dataset offers a comprehensive analysis of the underlying data concerning the complex interplay between Perceived organizational support (POS), job satisfaction (JS), work-life balance (WLB) and employee's intention to stay (ITS), with a specific focus on Vietnam's electronics industry. Notably, Vietnam has cemented its position as a leading exporter of electronic commodities, boasting exports exceeding USD 110 billion in 2023 [7]. These exports constitute approximately one-third of the nation's aggregate export value [7]. Concurrently, the industry's exponential growth necessitates a workforce surpassing 1 million labourers [8], a demand juxtaposed against a critical deficit of skilled labour, with 60 % of the sector's enterprises grappling with acute shortages [9].

POS is understood to be an employee's belief in the extent to which the organization appreciates their work and is concerned with their well-being [1]. Inoue and Alfaro‐Barrantes [10] defined ITS in an organization as the willingness to work continuously for the organization. WLB is the term used to describe how individuals perceive the harmony between their work responsibilities and personal life, and how these are aligned and adjusted according to their personal values, objectives, and ambitions [11]. Hoppock [12] defined Job satisfaction as the blend of mental, physical, and situational factors that lead someone to affirm honesty that they are content with their job.

This dataset is instrumental in dissecting how enterprises may enhance ITS in Vietnam's electronics industry. It scrutinizes the influence of POS on ITS and examines the mediating role of JS and WLB. Using a quantitative research approach, the dataset furnishes an exhaustive perspective on the pivotal determinants that shape ITS. These insights are invaluable for formulating nuanced policy-makers, elevating managerial practices, and improving the efficiency of the Vietnamese electronics industry.

## 3 Data Description

**Fig. 1:**
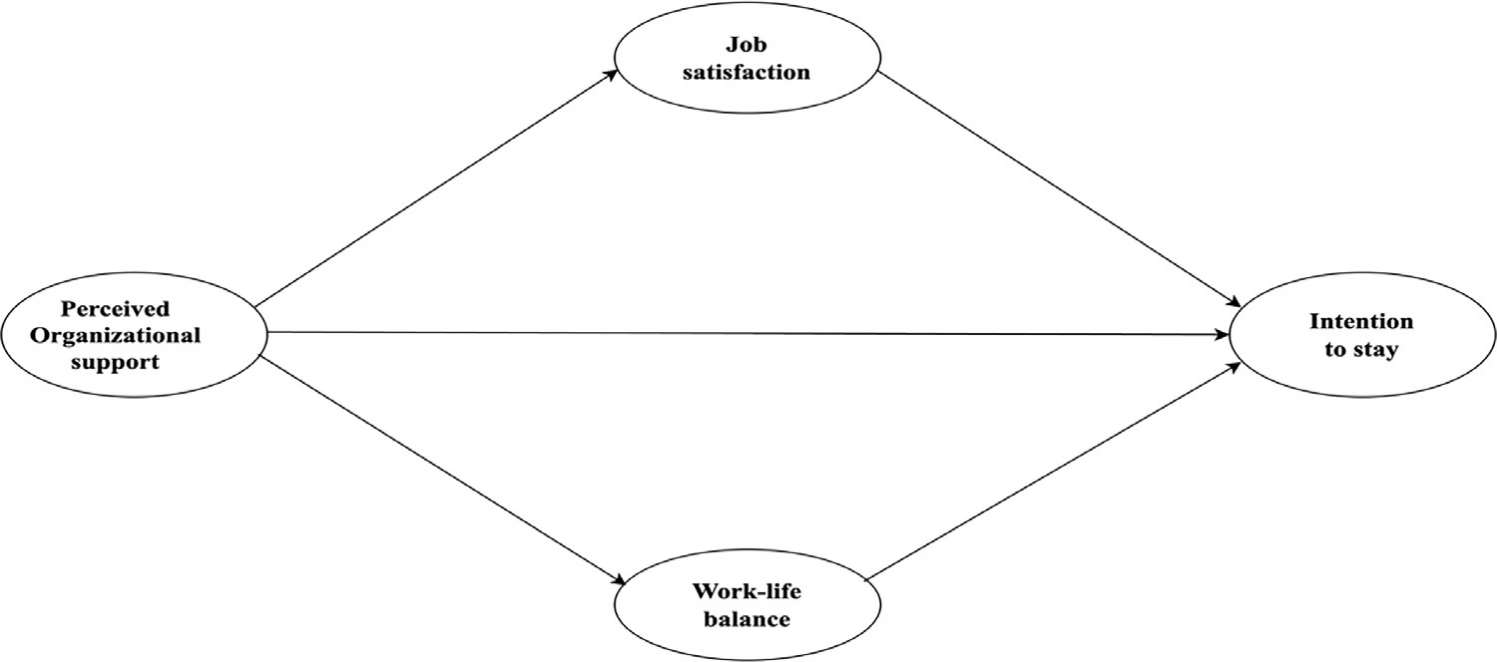
Conceptual model.

Between November 2022 and February 2024, a two-phase survey was implemented. Initially, a pilot survey was administered to 35 employees. Subsequently, a more comprehensive survey was carried out, yielding 604 responses that were deemed appropriate for analysis. The data repository is organized into two essential documents that clarify the data's nature and potential applications. The first document, named "Data-POS-ITS.csv", contains the numerical data collected from employee surveys. This file contains digital data collected from employees in the electronics industry in Vietnam. This file is organized in a manner conducive to statistical evaluation and theoretical modelling, containing 604 responses related to perceived organizational support (POS), job satisfaction (JS), Work-life balance (WLB) and Employee's Intention to Stay (ITS). The second crucial document, "Questionnaire.docx", comprises the comprehensive set of questions utilized during the data-gathering phase. The survey is designed to investigate four key areas: POS, JS, WLB and ITS, with ITS as the dependent variable, as shown in Fig. 1.

Based on previous research, each factor was precisely modified to align with the distinct characteristics of the current study. POS was measured using a scale adapted from Eisenberger, Huntington, Hutchison and Sowa [1]. This scale consisted of eight items. Job Satisfaction (JS) was quantified with a five-item metric based on Moqbel, Nevo and Kock [5]. Work-life balance (WLB) was assessed through a tri-item measure derived from Haar, Sune, Russo and Ollier-Malaterre [6]. Employee's Intention to Stay (ITS) was evaluated using a five-item instrument, which was constructed drawing upon the research of Bangwal and Tiwari [2], Mrayyan [3], and Nasyira, Othman and Ghazali [4]. All of the measures in this study leverage a five-point Likert scale. These measurement scales provide a detailed and sophisticated structure for examining the complex dynamics among different variables affecting ITS. The specific items from the questionnaire given to the respondents of this study are presented in Table 1.

**Table 1:** Measurement scale.

Construct	Item	Description
Employee's intention to stay (ITS)	ITS1	I am proud to be associated with the organization.
	ITS2	I intend to stay working at this organization for a long while.
	ITS3	It is very important for me to spend the rest of my career in this company.
	ITS4	I will stay at this company even if other companies offer me higher pay and position
	ITS5	Even if their job does not meet all of their expectations, they will not quit.

Job satisfaction (JS)	JS1	I am very satisfied with my current job.
	JS2	My present job gives me internal satisfaction.
	JS3	My job gives me a sense of fulfillment.
	JS4	I am very pleased with my current job.
	JS5	I will recommend this job to a friend if it is advertised/announced.

Perceived Organizational Support (POS)	POS1	The organization I work with values my contribution to its well-being.
	POS2	The organization fails to appreciate any extra effort from me.
	POS3	The organization would ignore any complaint from me.
	POS4	The organization really cares about my well-being.
	POS5	Even if I did the best job possible, the organization would fail to notice it.
	POS6	The organization cares about my general satisfaction at work.
	POS7	The organization shows very little concern for me.
	POS8	The organization takes pride in my accomplishments at work.

Work-life balance (WLB)	WLB1	I am satisfied with my work-life balance, enjoying both roles.
	WLB2	Nowadays, I seem to enjoy every part of my life equally well.
	WLB3	I manage to balance the demands of my work and personal life well.

Source(s): Author's work.

In the context of this study's linguistic requirements, the survey instrument was translated into Vietnamese with meticulous attention to detail, acknowledging the Vietnamese nationality of all participants. We utilized the back-translation methodology as advocated by Brislin [13] to maintain semantic equivalence between the languages. The process commenced with a bilingual specialist translating the survey from English to Vietnamese, followed by an independent expert who retranslated it into English. To ensure the precision and comprehensibility of the translated survey, a preliminary pilot was conducted using the Vietnamese iteration of the questionnaire.

**Fig. 2:**
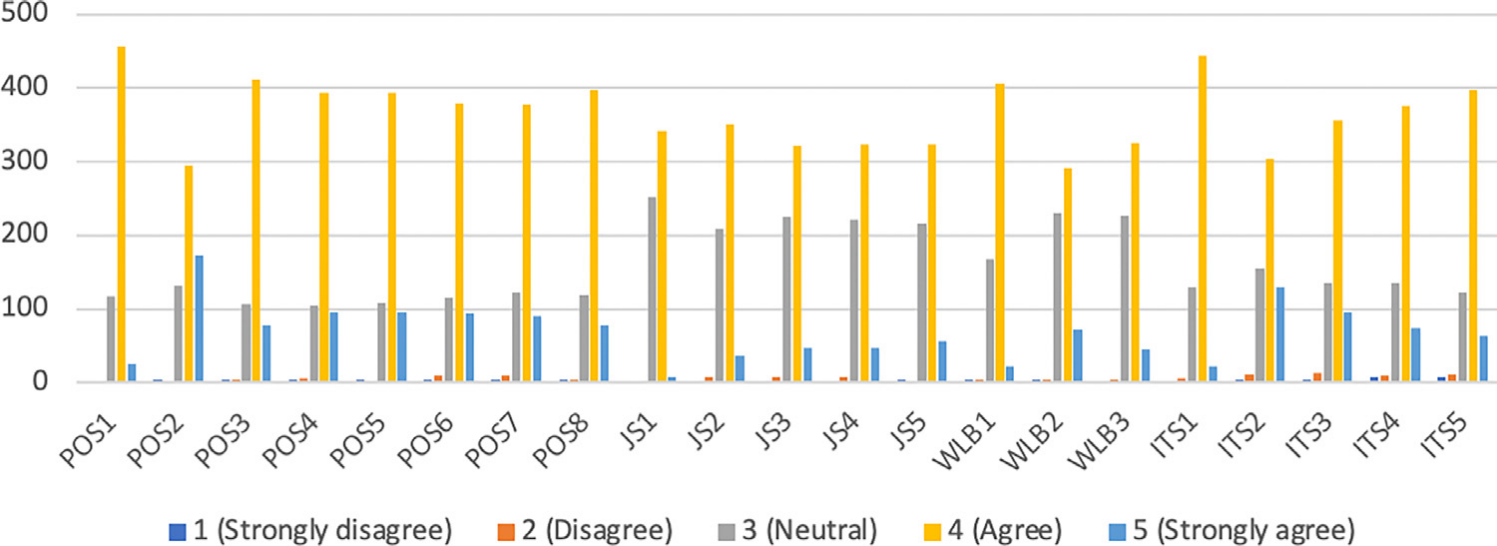
The distribution of respondent's answers.

Table 2 includes key statistical metrics such as Mean, Median, Minimum, Maximum, Standard Deviation, Excess Kurtosis, and Skewness. The means range from 3.575 to 4.038, indicating that respondents tend to rate closer to 4 on average on a scale from 1 to 5. Median is the middle value of the responses, which is 4 for all items, suggests that more than half of the respondents rated their agreement as 4 or higher. The minimum value is 1 for all items, showing that there is at least one respondent who rated the lowest possible score. The maximum value is 5 for all items, indicating that there is at least one respondent who rated the highest possible score. Standard deviation (SD) is A measure of variability in the responses. The SD values range from 0.511 to 0.779, which suggests that there is moderate variation in the responses for each item. Excess Kurtosis is a measure of the "tailedness" of the distribution. A positive kurtosis value indicates a distribution with heavier tails and sharper peak than the normal distribution. The values range from 0.372 to 4.566, with several items exhibiting high kurtosis, suggesting a concentration of responses at the extremes. Skewness is A measure of the asymmetry of the distribution of responses. Negative values indicate a left skew, meaning that there are more high-end responses (towards the maximum value of 5). The values range from −1.216 to −0.079, confirming the left skew in the data. Fig. 2 shows the distribution of respondent’s answers.

**Table 2:** Descriptive statistics of the constructs’ items.

Item	Mean	Median	Min	Max	Standard Deviation	Excess Kurtosis	Skewness
POS1	3.829	4	1	5	0.511	4.566	−1.216
POS2	4.038	4	1	5	0.761	0.423	−0.493
POS3	3.917	4	1	5	0.625	2.891	−0.796
POS4	3.944	4	1	5	0.654	2.357	−0.727
POS5	3.947	4	1	5	0.644	2.325	−0.66
POS6	3.904	4	1	5	0.691	2.1	−0.776
POS7	3.897	4	1	5	0.68	1.758	−0.66
POS8	3.889	4	1	5	0.648	2.703	−0.804
JS1	3.575	4	1	5	0.551	0.804	−0.594
JS2	3.677	4	1	5	0.625	0.999	−0.408
JS3	3.669	4	1	5	0.658	0.594	−0.186
JS4	3.674	4	1	5	0.657	0.624	−0.204
JS5	3.705	4	1	5	0.673	1.061	−0.287
WLB1	3.725	4	1	5	0.57	2.717	−0.999
WLB2	3.704	4	1	5	0.712	0.372	−0.079
WLB3	3.674	4	1	5	0.644	0.64	−0.166
ITS1	3.791	4	1	5	0.527	3.091	−1.129
ITS2	3.896	4	1	5	0.779	0.547	−0.469
ITS3	3.868	4	1	5	0.725	1.402	−0.656
ITS4	3.825	4	1	5	0.709	2.44	−0.909
ITS5	3.828	4	1	5	0.681	2.889	−1.031

Source(s): Author's work.

Table 3 outlines the statistical metrics for evaluating the constructs: POS, JS, WLB, and ITS. The outer loadings in the range of 0.747 to 0.852 indicate the strength of the correlation between each item and its respective construct. These values surpass the recommended threshold of 0.7 [14], affirming the items' contributions to measuring their underlying constructs. The Variance Inflation Factor (VIF) values, ranging between 1.498 and 2.389 [15], suggest slight correlations among predictors within constructs, but this does not pose a problem for the model. Cronbach's alpha (CA), used to assess the internal consistency or reliability of questionnaires, is reported in Table 3. Strong reliability is indicated with CA values exceeding 0.7 [16]. Additionally, composite reliability (CR), measuring internal consistency, exhibits values surpassing 0.8 [17], suggesting good internal consistency for all constructs. Average variance extracted (AVE) represents the amount of variance captured by the construct relative to measurement error. As indicated in Table 3, the AVE values for all constructs exceeded the threshold of 0.6, suggesting satisfactory convergent validity [14].

**Table 3:** Result of reliability and convergent validity.

	Outer loading	VIF	CA	CR	AVE
ITS1	0.811	1.933	0.879	0.912	0.674
ITS2	0.798	1.849			
ITS3	0.820	2.058			
ITS4	0.820	2.063			
ITS5	0.852	2.389			

JS1	0.783	1.767	0.850	0.893	0.625
JS2	0.747	1.566			
JS3	0.801	1.840			
JS4	0.833	2.073			
JS5	0.787	1.832			

POS1	0.757	1.951	0.911	0.928	0.617
POS2	0.774	2.100			
POS3	0.769	1.985			
POS4	0.790	2.085			
POS5	0.759	1.947			
POS6	0.818	2.322			
POS7	0.804	2.167			
POS8	0.810	2.166			

WLB1	0.843	1.525	0.759	0.861	0.674
WLB2	0.811	1.498			
WLB3	0.808	1.588			

Source(s): Author's work.

Fornell-Larcker criterion [18], as demonstrated in Table 4. This method involves comparing the square roots of the AVE with the inter-construct correlations, where our findings show the former exceeds the latter, thus establishing discriminant validity in line with the benchmarks set by Hair Jr, Sarstedt, Ringle and Gudergan [19]. Additionally, we conducted the Heterotrait-Monotrait (HTMT) ratio test, following the guidelines by Henseler, Ringle and Sarstedt [20], which suggest that for a robust model, HTMT values should be below 0.9 across all construct pairs. The data displayed in Table 5 adhere to this guideline with HTMT values under the threshold, reinforcing the discriminant validity of our research. These outcomes collectively affirm the robust reliability and validity of our study.

**Table 4:** Fornell-Larcker criterion.

	ITS	JS	POS	WLB
ITS	0.821			
JS	0.629	0.791		
POS	0.712	0.627	0.786	
WLB	0.626	0.681	0.525	0.821

**Source(s):** Author's work.

**Table 5:** Heterotrait-Monotrait Ratio (HTMT).

	ITS	JS	POS	WLB
ITS				
JS	0.726			
POS	0.790	0.705		
WLB	0.759	0.845	0.617	

Source(s): Author's work.

Table 6 presents the VIF values calculated from the inner model, a statistical instrument that quantifies the inflation in the variance of regression coefficients attributable to multicollinearity. Within the domain of raw data, VIF values provide a preliminary metric for assessing the inter-correlations among independent variables. The VIF metrics in this dataset fluctuate between 1.0 and 2.289. These values infer that the survey data's independent variables do not significantly enhance the variance of one another, signifying a multicollinearity extent that is low to moderate. This extent is considered tolerable within the scope of standard regression analytical practices [15]. Accordingly, this infers that the constructs exhibit a reasonable degree of independence in the data set.

**Table 6:** Collinearity statistic (VIF) – inner model.

	ITS	JS	POS	WLB
ITS				
JS	2.289			
POS	1.697	1.000		1.000
WLB	1.920			

Source(s): Author's work.

Table 7 outlines the direct and indirect effects between variables assessment, which was conducted using a bootstrapping method with 5000 resamples. The direct effects detail the connections between the studied factors, while the indirect effects elucidate how POS impacts ITS through JS and WLB. These statistical coefficients, calculated from the original dataset, demonstrate the relationship between variations in one variable and the consequent alterations in another, as reflected in the responses gathered from the survey. The findings from Table 7 indicate strong positive correlations between JS, POS, WLB and ITS (β = 0.139, *p* = 0.011; β = 0.478, *p* = 0.000; β = 0.281, *p* = 0.000, respectively). POS was significantly positively relationship with JS and WLB (β = 0.627, *p* = 0.000; β = 0.525, *p* = 0.000, respectively). POS influences ITS indirectly through JS (β = 0.087, *p* = 0.000). POS also affects ITS indirectly through WLB (β = 0.147, *p* = 0.000). In addition, Table 8 shows the total effect of endogenous variables on exogenous variables.

**Table 7:** Path analysis.

	Original Sample (O)	Sample Mean (M)	T Statistics (|O/STDEV|)	P Values
Direct effects				
JS -> ITS	0.139	0.139	2.547	0.011
POS -> ITS	0.478	0.477	8.086	0.000
POS -> JS	0.627	0.625	14.759	0.000
POS -> WLB	0.525	0.523	10.941	0.000
WLB -> ITS	0.281	0.283	5.995	0.000

Indirect effects				

POS -> JS -> ITS	0.087	0.087	2.486	0.013
POS -> WLB -> ITS	0.147	0.148	5.141	0.000

Source(s): Author's work.

**Table 8:** Total effect.

	Original Sample (O)	Sample Mean (M)	Standard Deviation (STDEV)	T Statistics (|O/STDEV|)	P Values
JS -> ITS	0.139	0.139	0.055	2.547	0.011
POS -> ITS	0.712	0.712	0.036	19.755	0.000
POS -> JS	0.627	0.625	0.042	14.759	0.000
POS -> WLB	0.525	0.523	0.048	10.941	0.000
WLB -> ITS	0.281	0.283	0.047	5.995	0.000

Source(s): Author's work.

Table 9 presents the f-square values that indicate the magnitude of influence that each independent variable exerts on a dependent variable. These f-square values are interpreted as measures of effect size (>=0.02 is small; >= 0.15 is medium;>= 0.35 is large) [21]. This data-driven metric sheds light on the quantitative significance of the independent variables in relation to the dependent variables within the collected dataset.

**Table 9:** f-square.

	ITS	JS	POS	WLB
ITS				
JS	0.021			
POS	0.339	0.646		0.381
WLB	0.103			

Source(s): Author's work.

**Fig. 3:**
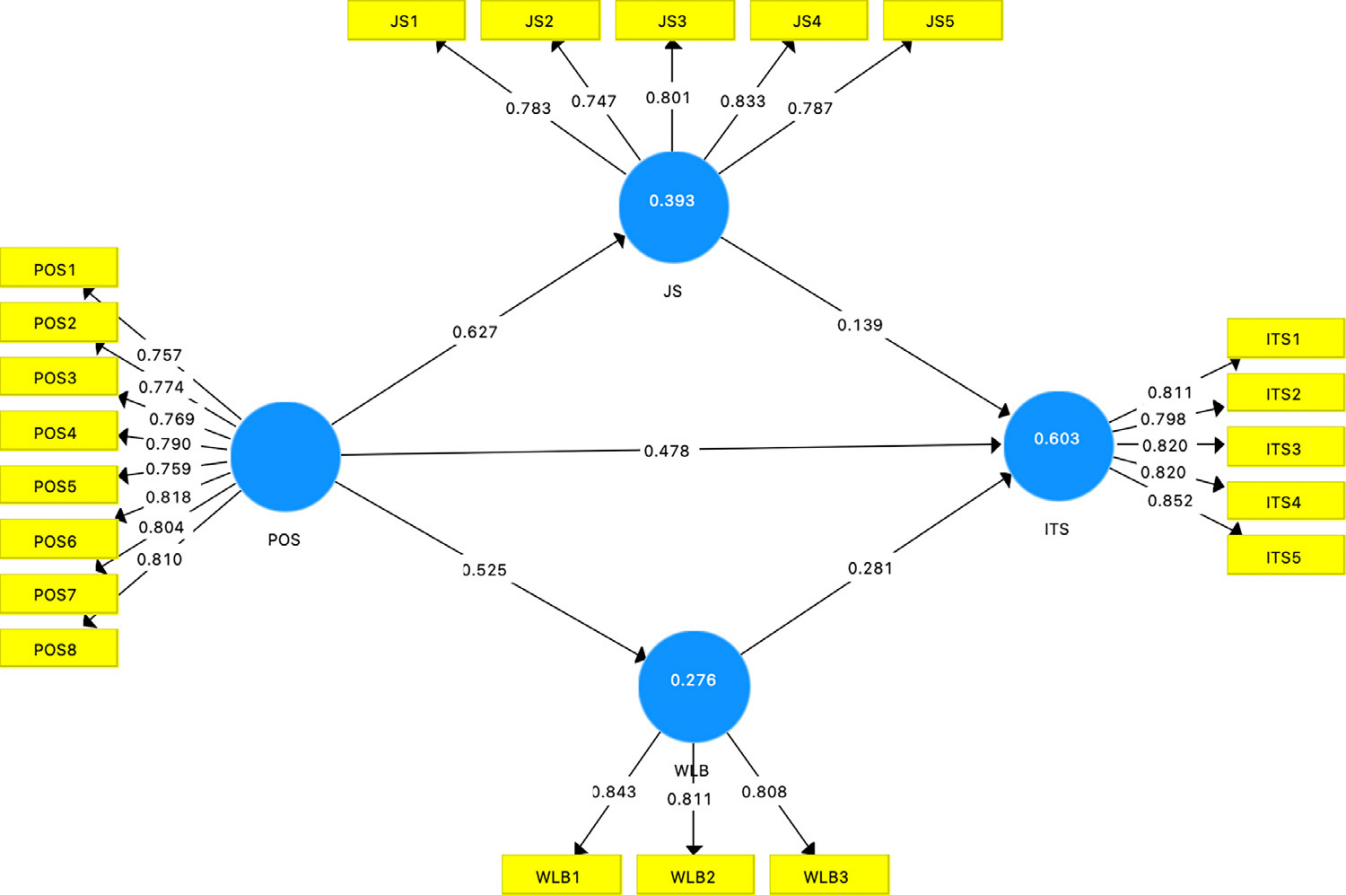
The PLS-SEM result.

Table 10 displays the Q-square (Q^2^) values, which assess the predictive relevance of a model, with values greater than 0 indicating a model's adequacy in prediction. The table shows that all Q^2^ values surpass this threshold, signifying that the model's endogenous constructs possess the anticipated predictive relevance [22]. Fig. 3 shows the measurement model's structure as applied to our dataset.

**Table 10:** Q-square.

	Q²
ITS	0.402
JS	0.241
POS	
WLB	0.179

Source(s): Author's work.

## 4 Experimental Design, Materials and Methods

Based on previous research, this study adopts a quantitative methodology. Data was collected through a structured survey questionnaire. An initial online pilot test involving 35 individuals evaluated the survey's structure and content. The structure and questions are assessed and modified based on their feedback to ensure effectiveness before data collection. The participants in the pre-test had no trouble understanding the questionnaire's questions, and there were no problems with the entire online process. The survey questionnaire consists of five parts: Demographics, Perceived Organizational Support (POS), Job Satisfaction (JS), Work-Life Balance (WLB) and Employee's intention to stay (ITS).

The questionnaire starts with collecting demographic data such as age, gender, marital status, monthly income, education, and job position. The second section probes into POS with eight specific items. The third section assesses JS through five items. The fourth section assesses WLB through three distinct items. The final section targets the ITS within the organization, evaluated through five items. Responses are measuring instruments on a five-point Likert scale, ranging from "strongly disagree (1)" to "strongly agree (5)."

Table 11 shows the demographic profiles of the survey paticipants, including variables age, gender, marital status, monthly income, education, and job position. The distribution of job positions among the participants is weighted towards workers, who constitute 54.97 % of the respondents. Executive Staff follows this at 25 %, Leaders at 8.77 %, Sub-leaders at 8.11 %, with the remaining 3.15 % falling into other categories. In terms of Working time at

**Table 11:** Demographic (*N* = 604).

Code	Characteristics	Categories	Frequency (*N* = 604)	Percentage (%)
Q1	Position	Worker	332	54.97
		Sub-leader	49	8.11
		Leader	53	8.77
		Executive Staff	151	25
		Other	19	3.15

Q2	Working time at the current company	Less than 02 years	239	39.57
		02–04 years	196	32.45
		05–07 years	131	21.69
		08–10 years	36	5.96
		> 10 years	02	0.33

Q3	The number of companies worked	Less than 2 companies	248	41.06
		2 - 4 Companies	277	45.86
		5 – 7 Companies	78	12.91
		8 – 10 Companies	01	0.17
		> 10 Companies	0	0

Q4	Age	18–27	375	62.09
		28–37	187	30.96
		38–46	42	6.95
		> 46	0	0

Q5	Gender	Male	163	26.99
		Female	441	73.01

Q6	Education	High school and below	326	53.97
		University/College	259	42.88
		Master and above	19	3.15

Q7	Monthly income	Less than $250	118	19.54
		$251-$400	256	42.38
		$401-$600	168	27.81
		> $601	62	10.26

Q8	Marital status	Single	310	51.32
		Married	294	48.68

*Note*: 1 USD, approximately 24,000 VND during the survey period.

The current company, a plurality (39.57 %) has been employed there for under two years. The subsequent groups are those with 2–4 years (32.45 %), 5–7 years (21.69 %), 8–10 years (5.96 %), and a negligible proportion (0.33 %) exceeding ten years.

Regarding the number of companies the respondents have worked for, 41.06 % have been with fewer than two companies and 45.86 % with 2–4 companies. A smaller fraction, 12.91 %, have worked at 5–7 companies, while an exceedingly small number, 0.17 %, have experience at 8–10 companies, and none have worked at more than ten companies.

The demographic data reveal a significant majority of females in the survey population, representing 73.01 %, and the age group most represented is the 18–27 year-olds, who account for 62.09 % of respondents. In educational attainment, the majority have a high school education or below (53.97 %), followed by those with a university/college degree (42.88 %), and a small segment with a master's degree and above (3.15 %). Income levels vary, with 19.54 % earning less than $250 monthly, 42.38 % earning between $251-$400, 27.81 % earning $401-$600, and 10.26 % earning more than $601. Regarding marital status, the respondents are nearly equally divided with 51.32 % being single and 48.68 % being married.

A methodical survey was constructed using Google Forms to solicit data from personnel employed within the electronics industry of Northern Vietnam. In Vietnam, foreign direct investment (FDI) accounts for the majority of funding for the electronics industry, usually accounting for 80–100 % of total investment capital. In particular, the Northern region stands out as the top destination for FDI, attracting about 78 % of large projects. Furthermore, it accounts for 81 % of total investment capital directed into Vietnam's electronics industry [23]. After the end of the survey, 604 legitimate responses were compiled from employees of 10 companies, including giants in the electronics industry such as Samsung, Luxshare, Foxcon, Khvatec. In particular, Samsung's production complexes in Thai Nguyen and Bac Ninh provinces contribute up to 60 % of the company's annual global output, making Vietnam Samsung's largest manufacturer [24]

This study used the Smart-PLS 3.0 application to analyse the data and evaluate the measurement model's validity. A meticulous and multifaceted statistical approach was applied during the data analysis to ensure the robustness and validity of the survey constructs. The relationship between items and their respective latent variables was examined by inspecting construct outer loadings, which is paramount in confirming the representational fidelity of each item. Cronbach's Alpha (CA) and Composite Reliability (CR) were computed for each construct to evaluate internal consistency and reliability. Furthermore, we incorporated the measurement of composite reliability for each construct to substantiate the reliability and internal consistency of the measurement scales.

The analysis of AVE substantiated that the majority of the variance observed in the constructs could be ascribed to their respective latent variables rather than to measurement error, thereby establishing convergent validity. In our efforts to preclude multicollinearity, the VIF was employed to confirm the distinctiveness of the constructs and their lack of excessive inter-correlations. We evaluated discriminant validity employing both the Fornell-Larcker criterion and the HTMT ratio. According to the Fornell-Larcker criterion, the square root of the AVE for each construct exceeds the construct's correlations, confirming stronger associations with its indicators compared to others. The HTMT ratio, a more rigorous and contemporary measure, was utilized to ascertain the constructs' uniqueness and verify that there was no significant overlap among them. By conducting this comprehensive analysis, we rigorously tested our theoretical framework, setting a solid groundwork for structural equation modelling (SEM) to explore the hypothesized relationships between constructs accurately.

## Limitations

Our study, which investigates Perceived Organizational Support (POS) and Employee's Intention to Stay (ITS) within Vietnam's electronics industry, faces several limitations. The applicability of this study's outcomes is circumscribed by the focus on a singular industry and cultural milieu, which may not be extrapolated directly to disparate industries or geographical locales.

## Ethics Statement

All researchers adhered to ethical guidelines throughout the study. Participants were thoroughly briefed on the objectives and scope of the research before their involvement. Informed consent was secured from each participant, who are presently unreachable. The study was conducted in a manner that did not require Institutional Review Board (IRB) approval. To safeguard participant anonymity, all collected data was stripped of personal identifiers and anonymized. Unique identification codes substituted any information that could potentially identify individuals, thereby maintaining the confidentiality and privacy of respondents in line with ethical standards for conducting research.

## CRediT authorship contribution statement

**Cong Hiep Duong:** Conceptualization, Methodology, Software, Validation, Formal analysis, Visualization, Writing – review & editing. **Yi-Hui Ho:** Conceptualization, Supervision, Methodology, Software, Data curation, Investigation, Writing – review & editing.

## Data Availability

Data-POS-ITS (Original data) (Mendeley Data).
